# Supplementary material for: Comprehensive Genetic and Molecular Characterization Confirms Hepatic Stellate Cell Origin of the Immortal Col-GFP HSC Line
Source: Int J Mol Sci. 2025 Aug 11;26(16):7764. doi: 10.3390/ijms26167764 (PMC12386721; doi:10.3390/ijms26167764)
Supplement: Supplementary file 1 [file ijms-26-07764-s001.zip › ijms-3744801-supplementary/Table 1-4 and 6-11.pdf]

International Journal of *Molecular Sciences*

Supplements

Comprehensive Genetic and Molecular Characterization Confirms Hepatic Stellate Cell Origin of the Immortal Col-GFP HSC Line

Larissa F. Buitkamp, Thomas Liehr, Stefanie Kankel, Eva Miriam Buhl, Katharina S. Hardt, Diandra T. Keller, Sarah K. Schröder-Lange and Ralf Weiskirchen

Table S1. Short tandem repeat (STR) profiling of Col-GFP HSC cells and comparison with other STR profiles of cell lines using CLASTR

				STR																	
**Accession**	**Name**	**Nº Markers**	**Score (%)**	**1-1**	**1-2**	**2-1**	**3-2**	**4-2**	**5-5**	**6-4**	**6-7**	**7-1**	**8-1**	**11-2**	**12-1**	**13-1**	**15-3**	**17-2**	**18-3**	**19-2**	**X-1**
**NA**	**Query**	**NA**	**NA**	**16**	**19**	**15**	**14**	**20.3**	**17**	**18**	**17**	**26.2,27.2**	**16**	**16**	**17**	**17**	**22.3**	**15**	**16,17**	**13**	**27**
CVCL_B7MI	Col-GFP HSC	18	100	16	19	15	14	20.3	17	18	17	26.2,27.2	16	16	17	17	22.3	15	16,17	13	27
CVCL_VR89	AT-3	18	82.93	16	19	16	14	20.3	17	18,19	15	26.2,27.2	16	16	17,18	17	22.3	15	16	13	27
CVCL_C8S6	OSUMMER.1	18	82.05	16	19	9	14	20.3	18	18	15	26.2,27.2	16	16	17	17	22.3	15	16	13	27
CVCL_C8SG	OSUMMER.11	18	82.05	16	19	9	14	20.3	18	18	15,17	26.2	16	16	17	17	22.3	15	16	13	27
CVCL_C8SH	OSUMMER.12	18	82.05	16	19	9	14	20.3	18	18	15,17	26.2	16	16	17	17	22.3	15	16	13	27
CVCL_C8S7	OSUMMER.2	18	82.05	16	19	9	14	20.3	18	18	15	26.2,27.2	16	16	17	17	22.3	15	16	13	27
CVCL_JK10	YUMM1.1	18	82.05	16,17	19	16	14	20.3	17	18	17	27.2	16	16	17	17	22.3	16	16	13	27
CVCL_JK12	YUMM1.3	18	82.05	16,17	19	16	14	20.3	17	18	17	27.2	16	16	17	17	22.3	16	16	13	27
CVCL_JK38	YUMM4.1	18	82.05	16,17	19	16	14	20.3	17	18	17	27.2	16	16	17	17	22.3	16	16	13	27
CVCL_C8SC	OSUMMER.7	18	80.95	16	19	9,16	14	20.3	18,19	18	15	26.2,27.2	16	16	17	17	22.3	15	16,17	13	27
CVCL_C8SI	OSUMMER.13	18	80.00	16	19	9	14	20.3	18	18	15,17	26.2	16	16	17,18	17	22.3	15	16	13	27
CVCL_C8SB	OSUMMER.6	18	80.00	16	19	9	14	20.3	18	18	15	26.2,27.2	16	16	17	17	22.3	15	16	13	26,27
CVCL_C8SE	OSUMMER.9	18	80.00	16	19	9,16	14	20.3	18	18	15	26.2,27.2	16	16	17	17	22.3	15	16	13	27
CVCL_A2AY	YUMMER1.7 H2B-GFP5	18	80.00	16,17	19	16,17	14	20.3	17	18	17	27.2	16	16	17	17	22.3	16	16	13	27

The search was conducted using the Cellosaurus STR Similarity Search Tool CLASTR1.4.4 with the following parameters: Algorithm: Tanabe; Modes: Non-empty markers; Score filter: 60%; Min. Markers: 8. Allelic markers that differ between the Col-GFP HSCs and other cells are marked in red.

International Journal of
Molecular Sciences

Table S2. Features and sources of murine cell lines

Cell line	Cellular origin	Source of immortalized cell line	Reference
Col-GFP HSC	Hepatic stellate cell (liver)	C57BL/6	[1]
AT-3	Mammary gland carcinoma (breast)	B6.FVB-Tg(MMTV-PyVT)634Mul/LellJ (MTAG)	[2]
OSUMMER.1	Melanoma (skin)	C57BL/6 TN61R (Tyr::CreER(T2)-driven, Nras)	[3]
OSUMMER.11	Melanoma (skin)	C57BL/6 TN61R (Tyr::CreER(T2)-driven, Nras)	[3]
OSUMMER.12	Melanoma (skin)	C57BL/6 TN61R (Tyr::CreER(T2)-driven, Nras)	[3]
OSUMMER.2	Melanoma (skin)	C57BL/6 TN61R (Tyr::CreER(T2)-driven, Nras)	[3]
YUMM1.1	Melanoma (skin)	C57BL/6	[4]
YUMM1.3	Melanoma (skin)	C57BL/6	[4]
YUMM4.1	Melanoma (skin)	C57BL/6	[4]
OSUMMER.7	Melanoma (skin)	C57BL/6 TN61R (Tyr::CreER(T2)-driven, Nras)	[3]
OSUMMER.13	Melanoma (skin)	C57BL/6 TN61R (Tyr::CreER(T2)-driven, Nras)	[3]
OSUMMER.6	Melanoma (skin)	C57BL/6 TN61R (Tyr::CreER(T2)-driven, Nras)	[3]
OSUMMER.9	Melanoma (skin)	C57BL/6 TN61L (Tyr::CreER(T2)-driven, Nras)	[3]
YUMMER1.7 H2B-GFP5	YUMMER1.7 H2B-GFP5	C57BL/6	[4,5]

International Journal of
Molecular Sciences

Table S3. Short tandem repeat (STR) profiling of AML12 cells and comparison with other STR profiles of cell lines using CLASTR

				STR																	
**Accession**	**Name**	**Nº Markers**	**Score (%)**	**1-1**	**1-2**	**2-1**	**3-2**	**4-2**	**5-5**	**6-4**	**6-7**	**7-1**	**8-1**	**11-2**	**12-1**	**13-1**	**15-3**	**17-2**	**18-3**	**19-2**	**X-1**
**NA**	**Query**	**NA**	**NA**	**11**	**13**	**9**	**12**	**20.3**	**14,15**	**15.3**	**12**	**29**	**14,15**	**18**	**19**	**15**	**21.3**	**13,15**	**21**	**13**	**26**
CVCL_0140	AML12	18	88.8	NN	NN	NN	NN	20.3	14,15	16	12	NN	NN	NN	19	NN	21.3	NN	21	NN	26

The search was conducted using the Cellosaurus STR Similarity Search Tool CLASTR1.4.4 with the following parameters: Algorithm: Tanabe; Modes: Non-empty markers; Score filter: 60%; Min. Markers: 8. Allelic markers that differ between the tested AML12 cells and information depicted in the entry for CVCL_0140 are marked in red. NN, not known. Please note that only eight STR markers for AML12 (entry CVCL_0140) are currently deposited in the Cellosaurus reference bank (as of 24 July 2025).

International Journal of
Molecular Sciences

Table S4. Selected gene expression in Col-GFP HSCs that underpins their fibrogenic origin

Gene	Gene description	Gene Id	Transcript Id	TPM[1]
Acta2	actin alpha 2, smooth muscle, aorta	ENSMUSG00000035783	ENSMUST00000039631.10	12.213945
Bgn	biglycan	ENSMUSG00000031375	ENSMUST00000033741.15	32.306801
Ccl2	chemokine (C-C motif) ligand 2	ENSMUSG00000035385	ENSMUST00000000193.6	21.31315
Ccn1	cellular communication network factor 1	ENSMUSG00000028195	ENSMUST00000029846.5	311.16279
Ccn2	cellular communication network factor 2	ENSMUSG00000019997	ENSMUST00000020171.12	120.652872
Col1a1	collagen, type I, alpha 1	ENSMUSG00000001506	ENSMUST00000001547.8	32.542562
Col1a2	collagen, type I, alpha 2	ENSMUSG00000029661	ENSMUST00000031668.10	6.678061
Col3a1	collagen, type III, alpha 1	ENSMUSG00000026043	ENSMUST00000087883.13	45.359998
Colec11	collectin sub-family member 11	ENSMUSG00000036655	ENSMUST00000036136.9	0.358516
Csrp2	cysteine and glycine-rich protein 2	ENSMUSG00000020186	ENSMUST00000020403.7	19.252597
Cygb	cytoglobin	ENSMUSG00000020810	ENSMUST00000021166.6	0.412708
Dcn	decorin	ENSMUSG00000019929	ENSMUST00000105287.11	78.633523
Des	desmin	ENSMUSG00000026208	ENSMUST00000027409.10	0.155645
Eng	endoglin	ENSMUSG00000026814	ENSMUST00000009705.14	24.094436
			ENSMUST00000113272.8	20.652491
			ENSMUST00000167841.8	2.10756
Fgf10	fibroblast growth factor 10	ENSMUSG00000021732	ND	0
Gfap	glial fibrillary acidic protein	ENSMUSG00000020932	ND	0
Hgf	hepatocyte growth factor	ENSMUSG00000028864	ND	0
Igfbp6	insulin-like growth factor binding protein 6	ENSMUSG00000023046	ENSMUST00000023807.7	28.779598
Igfbp7	insulin-like growth factor binding protein 7	ENSMUSG00000036256	ENSMUST00000046746.103	5.253428
Ltbp1	latent transforming growth factor beta binding protein 1	ENSMUSG00000001870	ENSMUST00000112516.8	19.219672
Ltbp2	latent transforming growth factor beta binding protein 2	ENSMUSG00000002020	ENSMUST00000163189.8	0.140632
Ltbp3	latent transforming growth factor beta binding protein 3	ENSMUSG00000024940	ENSMUST00000081496.6	27.556623
Ltbp4	latent transforming growth factor beta binding protein 4	ENSMUSG00000040488	ENSMUST00000121175.8	7.617136
Ngfr	nerve growth factor receptor	ENSMUSG00000000120	ND	0
Ppara	peroxisome proliferator activated receptor alpha	ENSMUSG00000022383	ND	0
Pdgfra	platelet derived growth factor receptor, alpha polypeptide	ENSMUSG00000029231	ENSMUST00000000476.15	0.24807
			ENSMUST00000202681.4	0.234793
Pparg	peroxisome proliferator activated receptor gamma	ENSMUSG00000000440	ENSMUST00000203732.3	8.052454
			ENSMUST00000171644.8	0.956455
Rbp1	retinol binding protein 1, cellular	ENSMUSG00000046402	ENSMUST00000052068.11	0.179431
Reln	reelin	ENSMUSG00000042453	ND	0
Sparc	secreted acidic cysteine rich glycoprotein	ENSMUSG00000018593	ENSMUST00000108858.8	511.003164

			ENSMUST00000108858.8	437.359887
			ENSMUST00000018737.13	17.829097
Syp	synaptophysin	ENSMUSG00000031144	ND	0
Tagln	transgelin	ENSMUSG00000032085	ENSMUST00000034590.4	8.808124
Tgfa	transforming growth factor alpha	ENSMUSG00000029999	ENSMUST00000032066.13	1.120124
Tgfb1i1	transforming growth factor beta 1 induced transcript 1	ENSMUSG00000030782	ENSMUST00000167965.8	1.136066
Tgfbrap1	transforming growth factor, beta receptor associated protein 1	ENSMUSG00000070939	ENSMUST00000186694.7	12.748725
			ENSMUST00000001927.12	2.832279
			ENSMUST00000095014.8	1.143414
Tbrg1	transforming growth factor beta regulated gene 1	ENSMUSG00000011114	ENSMUST00000117654.3	85.713616
Tbrg4	transforming growth factor beta regulated gene 4	ENSMUSG00000000384	ENSMUST00000000394.14	81.416015
			ENSMUST00000189268.7	26.834916
Tgfb1	transforming growth factor, beta 1	ENSMUSG00000002603	ENSMUST00000002678.10	7.56787
Tgfb2	transforming growth factor, beta 2	ENSMUSG00000039239	ENSMUST00000045288.14	8.726819
			ENSMUST00000195201.2	3.171098
Tgfb3	transforming growth factor, beta 3	ENSMUSG00000021253	ENSMUST00000003687.8	2.628305
Tgfbi	transforming growth factor, beta induced	ENSMUSG00000035493	ENSMUST00000045173.10	4.200504
Tgfbr1	transforming growth factor, beta receptor I	ENSMUSG00000007613	ENSMUST00000007757.15	12.349202
			ENSMUST00000107725.3	0.446599
			ENSMUST00000044234.14	0.29915
Tgfbr2	transforming growth factor, beta receptor II	ENSMUSG00000032440	ENSMUST00000035014.8	7.962054
			ENSMUST00000061101.12	7.30709
Tgfbr3	transforming growth factor, beta receptor III	ENSMUSG00000029287	ENSMUST00000031224.15	6.589789
Timp1	tissue inhibitor of metalloproteinase 1	ENSMUSG00000001131	ENSMUS00000009530.5	57.375035
			ENSMUST00000115342.10	52.129685
Tpm2	tropomyosin 2, beta	ENSMUSG00000028464	ENSMUST00000107914.10	44.234264
			ENSMUST00000107913.10	8.237037
Vcl	vinculin	ENSMUSG00000021823	ENSMUST00000022369.9	36.010773
Vegfa	vascular endothelial growth factor A	ENSMUSG00000023951	ENSMUST00000071648.12	38.271239
			ENSMUST00000024747.14	38.072879
			ENSMUST00000142351.9	3.843999
Wt1	WT1 transcription factor	ENSMUSG00000016458	ENSMUST00000143043.8	8.431285
			ENSMUST00000111099.2	6.939276
			ENSMUST00000111098.8	5.066908
Gapdh	glyceraldehyde-3-phosphate dehydrogenase	ENSMUSG00000057666	ENSMUST00000118875.8	4856.813242
			ENSMUST00000117757.9	2833.490407
			ENSMUST00000073605.15	813.747632

[1] For the comparison of transcript levels of the listed genes, the expression of the housekeeping gene glyceraldehyde-3-phosphate dehydrogenase (*Gapdh*) is shown. The complete mRNA expression profile of Col-GFP HSC cells, as observed by NGS, can be found in Table S5. ND indicates that no transcripts of this gene were detected. TPM, Transcripts Per Million.

International Journal of
Molecular Sciences

Table S5. Gene expression in Col-GFP HSCs as assessed by Next Generation Sequencing

REMARK: For this table, please refer to separte file

International Journal of
Molecular Sciences

Table S6. Selected hepatocyte marker gene expression in Col-GFP HSCs

Gene	Gene description	**Gene Id**	**Transcript Id**	**TPM**[1]
A1cf	APOBEC1 complementation factor	ENSMUSG00000052595	ND	0
Abcc3	ATP-binding cassette, sub-family C (CFTR/MRP), member 3	ENSMUSG00000020865	ENSMUST00000021231.8	0.091832
Abcd3	ATP-binding cassette, sub-family D (ALD), member 3	ENSMUSG00000028127	ENSMUST00000029770.8	34.27454
Acadm	acyl-Coenzyme A dehydrogenase, medium chain	ENSMUSG00000062908	ENSMUST00000072697.13	52.58478
Acat2	acetyl-Coenzyme A acetyltransferase 2	ENSMUSG00000023832	ENSMUST00000007005.14<br>ENSMUST00000233792.2<br>ENSMUST00000159697.2	111.213456<br>16.422877<br>10.088342
Acly	ATP citrate lyase	ENSMUSG00000020917	ENSMUST00000107389.8<br>ENSMUST00000165111.8	138.949765<br>74.303793
Acss2	acyl-CoA synthetase short-chain family member 2	ENSMUSG00000027605	ENSMUST00000029135.15	8.380149
Afp	alpha fetoprotein	ENSMUSG00000054932	ND	0
Alb	albumin	ENSMUSG00000029368	ENSMUST00000031314.10	2.156572
Aldh6a1	aldehyde dehydrogenase family 6, subfamily A1	ENSMUSG00000021238	ENSMUST00000085192.7	15.691162
Ahr	aryl-hydrocarbon receptor	ENSMUSG00000019256	ENSMUST00000116436.9	10.384841
Ambp	alpha 1 microglobulin/bikunin precursor	ENSMUSG00000028356	ENSMUST00000030041.5	0.410143
Ang	angiogenin, ribonuclease, RNase A family, 5	ENSMUSG00000072115	ENSMUST00000171688.9<br>ENSMUST00000069011.9	3.269515<br>0.605759
Anxa13	annexin A13	ENSMUSG00000055114	ND	0
Apoa1	apolipoprotein A-I	ENSMUSG00000032083	ENSMUST00000034588.9	0.523453
Apoa2	apolipoprotein A-II	ENSMUSG00000005681	ND	0
Apoa5	apolipoprotein A-V	ENSMUSG00000032079	ND	0
Apob	apolipoprotein B	ENSMUSG00000020609	ENSMUST00000037811.13	0.032125
Apoc3	apolipoprotein C-III	ENSMUSG00000032081	ND	0
Apoh	apolipoprotein H	ENSMUSG00000000049	ND	0
Apom	apolipoprotein M	ENSMUSG00000024391	ND	0
Aqp9	aquaporin 9	ENSMUSG00000032204	ENSMUST00000113570.8	0.290368
Ar	androgen receptor	ENSMUSG00000046532	ENSMUST00000052837.9	0.627063
Arg1	arginase, liver	ENSMUSG00000019987	ND	0
Asgr1	asialoglycoprotein receptor 1	ENSMUSG00000020884	ND	0
Asl	argininosuccinate lyase	ENSMUSG00000025533	ENSMUST00000161094.8<br>ENSMUST00000159619.8	25.299534<br>1.96766
Ass1	argininosuccinate synthetase 1	ENSMUSG00000076441	ENSMUST00000102840.5	117.039955
Atp7b	ATPase, Cu++ transporting, beta polypeptide	ENSMUSG00000006567	ENSMUST00000006742.11	0.139852
Azgp1	alpha-2-glycoprotein 1, zinc	ENSMUSG00000037053	ND	0
Bche	butyrylcholinesterase	ENSMUSG00000027792	ENSMUST00000029367.6	0.073354
Bnip3	BCL2/adenovirus E1B interacting protein 3	ENSMUSG00000078566	ENSMUST00000106112.2	112.598161

International Journal of
Molecular Sciences

C4b	complement component 4B (Chido blood group)	ENSMUSG00000073418	ND	0
Cdh1	cadherin 1	ENSMUSG00000000303	ND	0
Cebpa	CCAAT/enhancer binding protein (C/EBP), alpha	ENSMUSG00000034957	ND	0
Cp	ceruloplasmin	ENSMUSG00000003617	ENSMUST00000091309.12<br>ENSMUST00000003714.13	0.314981<br>0.142852
Cps1	carbamoyl-phosphate synthetase 1	ENSMUSG00000025991	ENSMUST00000027144.8	0.082312
Crp	C-reactive protein, pentraxin-related	ENSMUSG00000037942	ND	0
Ctnnb1	catenin (cadherin associated protein), beta 1	ENSMUSG00000006932	ENSMUST00000007130.15<br>ENSMUST00000178812.9	302.575732<br>1.26441
Cyp1a1	cytochrome P450, family 1, subfamily a, polypeptide 1	ENSMUSG00000032315	ND	0
Cyp1a2	cytochrome P450, family 1, subfamily a, polypeptide 2	ENSMUSG00000032310	ND	0
Cyp2e1	cytochrome P450, family 2, subfamily e, polypeptide 1	ENSMUSG00000025479	ND	0
Cyp2f2	cytochrome P450, family 2, subfamily f, polypeptide 2	ENSMUSG00000052974	ND	0
Cyp7a1	cytochrome P450, family 7, subfamily a, polypeptide 1	ENSMUSG00000028240	ENSMUST00000029905.2	0
Defb1	defensin beta 1	ENSMUSG00000044748	ND	0
Eid2	EP300 interacting inhibitor of differentiation 2	ENSMUSG00000046058	ENSMUST00000059596.8	11.401017
Entpd5	ectonucleoside triphosphate diphosphohydrolase 5	ENSMUSG00000021236	ENSMUST00000110272.9<br>ENSMUST00000072061.12	5.773825<br>4.302612
Epb41l4b	erythrocyte membrane protein band 4.1 like 4b	ENSMUSG00000028434	ENSMUST00000030142.4	1.011425
Eppk1	epiplakin 1	ENSMUSG00000118671	ENSMUST00000239552.1	0.109091
Fcna	ficolin A	ENSMUSG00000026938	ND	0
Fga	fibrinogen alpha chain	ENSMUSG00000028001	ENSMUST00000029630.15	0.700234
Fgfr4	fibroblast growth factor receptor 4	ENSMUSG00000005320	ND	0
Fgg	fibrinogen gamma chain	ENSMUSG00000033860	ENSMUST00000048486.13	0.293651
Fgl1	fibrinogen-like protein 1	ENSMUSG00000031594	ENSMUST00000034003.5	0.475527
Fosl1	fos-like antigen 1	ENSMUSG00000024912	ENSMUST00000025850.7	56.755003
Foxa1	forkhead box A1	ENSMUSG00000035451	ND	0
Foxa2	forkhead box A2	ENSMUSG00000037025	ENSMUST00000109964.8	0.235027
Foxa3	forkhead box A3	ENSMUSG00000040891	ND	0
Fst	follistatin	ENSMUSG00000021765	ENSMUST00000231252.2	0.479139
G0s2	G0/G1 switch gene 2	ENSMUSG00000009633	ND	0
G6pc	glucose-6-phosphatase, catalytic	ENSMUSG00000078650	ND	0
Gc	vitamin D binding protein	ENSMUSG00000035540	ENSMUST00000049209.13	0.27431
Gck	glucokinase	ENSMUSG00000041798	ND	0
Ghr	growth hormone receptor	ENSMUSG00000055737	ENSMUST00000069451.11<br>ENSMUST00000110698.9	8.108821<br>6.235911
Gjb2	gap junction protein, beta 2	ENSMUSG00000046352	ND	0

Gls2	glutaminase 2 (liver, mitochondrial)	ENSMUSG00000044005	ENSMUST00000044776.13	10.752265
Glul	glutamate-ammonia ligase (glutamine synthetase)	ENSMUSG00000026473	ENSMUST00000086199.12	22.240019
Golt1a	golgi transport 1A	ENSMUSG00000103421	ND	0
Gpat4	glycerol-3-phosphate acyltransferase 4	ENSMUSG00000031545	ENSMUST00000167004.3	69.37356
Gpc3	glypican 3	ENSMUSG00000055653	ENSMUST00000069360.14	1.526535
Grp	gastrin releasing peptide	ENSMUSG00000024517	ND	0
Grb14	growth factor receptor bound protein 14	ENSMUSG00000026888	ENSMUST00000028252.14	1.722203
Gsta2	glutathione S-transferase, alpha 2 (Yc2)	ENSMUSG00000057933	ND	0
Gulo	gulonolactone (L-) oxidase	ENSMUSG00000034450	ND	0
Hal	histidine ammonia lyase	ENSMUSG00000020017	ENSMUST00000129421.8	0.0861
Hhex	hematopoietically expressed homeobox	ENSMUSG00000024986	ENSMUST00000025944.9	9.869037
Hmgcs1	3-hydroxy-3-methylglutaryl-Coenzyme A synthase 1	ENSMUSG00000093930	ENSMUST00000179869.3	163.18192
			ENSMUST00000224188.2	10.707405
Hnf4a	hepatic nuclear factor 4, alpha	ENSMUSG00000017950	ND	0
Hsd11b1	hydroxysteroid 11-beta dehydrogenase 1	ENSMUSG00000016194	ND	0
Kif13b	kinesin family member 13B	ENSMUSG00000060012	ENSMUST00000224503.2	2.035974
Lepr	leptin receptor	ENSMUSG00000057722	ENSMUST00000102777.10	0.137171
Lrp5	low density lipoprotein receptor-related protein 5	ENSMUSG00000024913	ENSMUST00000025856.17	22.920188
Masp2	mannan-binding lectin serine peptidase 2	ENSMUSG00000028979	ND	0
Mcc	mutated in colorectal cancers	ENSMUSG00000071856	ENSMUST00000089874.9	9.862569
			ENSMUST00000164666.6	1.904627
Mrps18c	mitochondrial ribosomal protein S18C	ENSMUSG00000016833	ENSMUST00000016977.15	105.285666
			ENSMUST00000112901.2	1.047141
Msmo1	methylsterol monoxygenase	ENSMUSG00000031604	ENSMUST00000034015.11	179.93578
Mup3	major urinary protein 3	ENSMUSG00000066154	ND	0
Nnt	nicotinamide nucleotide transhydrogenase	ENSMUSG00000116207	ENSMUST00000223268.2	29.172993
			ENSMUST00000069902.13	16.761917
			ENSMUST00000109204.8	0.257591
Nos2	nitric oxide synthase 2, inducible	ENSMUSG00000020826	ND	0
Nr1i3	nuclear receptor subfamily 1, group I, member 3	ENSMUSG00000005677	ND	0
Ociad1	OCIA domain containing 1	ENSMUSG00000029152	ENSMUST00000031038.11	176.64486
			ENSMUST00000202250.4	49.571359
			ENSMUST00000166823.5	2.50425
			ENSMUST00000071081.13	1.1915
Orm1	orosomucoid 1	ENSMUSG00000039196	ND	0
Otc	ornithine transcarbamylase	ENSMUSG00000031173	ND	0
Pah	phenylalanine hydroxylase	ENSMUSG00000020051	ND	0
Pck1	phosphoenolpyruvate carboxykinase 1, cytosolic	ENSMUSG00000027513	ND	0
Phlda1	pleckstrin homology like domain, family A, member 1	ENSMUSG00000020205	ENSMUST00000164773.2	28.637812
Plin1	perilipin 1	ENSMUSG00000030546	ND	0
Plscr1	phospholipid scramblase 1	ENSMUSG00000032369	ENSMUST00000186364.2	23.587894

			ENSMUST00000093801.10	3.062996
Pon3	paraoxonase 3	ENSMUSG00000029759	ENSMUST00000031773.9	5.097862
Prox1	prospero homeobox 1	ENSMUSG00000010175	ND	0
Prrg4	proline rich Gla (G-carboxyglutamic acid) 4 (transmembrane)	ENSMUSG00000027171	ENSMUST00000028593.11	1.976449
Rcan1	regulator of calcineurin 1	ENSMUSG00000022951	ENSMUST00000232239.2	20.866982
			ENSMUST00000023672.10	10.039403
Rhob	ras homolog family member B	ENSMUSG00000054364	ENSMUST00000067384.6	115.20794
Rnd3	Rho family GTPase 3	ENSMUSG00000017144	ENSMUST00000017288.9	61.074753
			ENSMUST00000154545.2	0.202338
Rpp25l	ribonuclease P/MRP 25 subunit-like	ENSMUSG00000036114	ENSMUST00000038434.4	57.144178
Saa4	serum amyloid A 4	ENSMUSG00000040017	ND	0
Samd5	sterile alpha motif domain containing 5	ENSMUSG00000060487	ENSMUST00000100070.5	0.066623
Sat2	spermidine/spermine N1-acetyl transferase 2	ENSMUSG00000069835	ENSMUST00000108656.9	5.686217
Scd1	stearoyl-Coenzyme A desaturase 1	ENSMUSG00000037071	ENSMUST00000041331.4	92.94814
Sdc2	syndecan 2	ENSMUSG00000022261	ENSMUST00000022871.7	20.912812
Sec16b	SEC16 homolog B, endoplasmic reticulum export factor	ENSMUSG00000026589	ENSMUST00000111700.8	0.211362
Serpina1c	serine (or cysteine) peptidase inhibitor, clade A, member 1C	ENSMUSG00000079015	ND	0
Serpinh1	serine (or cysteine) peptidase inhibitor, clade H, member 1	ENSMUSG00000070436	ENSMUST00000094154.6	411.662825
			ENSMUST00000207849.2	299.707162
			ENSMUST00000169437.9	41.478724
Serpina6	serine (or cysteine) peptidase inhibitor, clade A, member 6	ENSMUSG00000060807	ND	0
Sfrp5	secreted frizzled-related sequence protein 5	ENSMUSG00000018822	ND	0
Slbp	stem-loop binding protein	ENSMUSG00000004642	ENSMUST00000057551.14	148.603745
			ENSMUST00000139518.8	2.173281
Slc2a2	solute carrier family 2 (facilitated glucose transporter), member 2	ENSMUSG00000027690	ND	0
Slc10a1	solute carrier family 10 (sodium/bile acid cotransporter family), member 1	ENSMUSG00000021135	ND	0
Slpi	secretory leukocyte peptidase inhibitor	ENSMUSG00000017002	ND	0
Sptbn1	spectrin beta, non-erythrocytic 1	ENSMUSG00000020315	ENSMUST00000011877.13	68.455691
			ENSMUST00000006629.14	23.136164
			ENSMUST00000102838.10	15.148833
Sult1a1	sulfotransferase family 1A, phenol-preferring, member 1	ENSMUSG00000030711	ND	0
Tat	tyrosine aminotransferase	ENSMUSG00000001670	ND	0
Tfr2	transferrin receptor 2	ENSMUSG00000029716	ND	0
Tkfc	triokinase, FMN cyclase	ENSMUSG00000034371	ENSMUST00000037678.7	11.564753
Tm4sf4	transmembrane 4 superfamily member 4	ENSMUSG00000027801	ENSMUST00000029377.8	1.692258

Tm4sf5	transmembrane 4 superfamily member 5	ENSMUSG00000018919	ND	0
Tm7sf2	transmembrane 7 superfamily member 2	ENSMUSG00000024799	ENSMUST00000025713.12	5.911766
			ENSMUST00000161528.2	1.052914
			ENSMUST00000113543.9	0.871438
Tmem97	transmembrane protein 97	ENSMUSG00000037278	ENSMUST00000103242.5	188.391346
Ttc36	tetratricopeptide repeat domain 36	ENSMUSG00000039438	ND	0
Ttr	transthyretin	ENSMUSG00000061808	ENSMUST00000075312.5	0.443965
Ucp2	uncoupling protein 2 (mitochondrial, proton carrier)	ENSMUSG00000033685	ENSMUST00000126534.8	1.976878
Ugt1a1	UDP glucuronosyltransferase 1 family, polypeptide A1	ENSMUSG00000089960	ND	0
Wtap	WT1 associating protein	ENSMUSG00000060475	ENSMUST00000007007.14	60.679582
			ENSMUST00000160781.8	50.732039
			ENSMUST00000159986.8	3.363958
Wt1	WT1 transcription factor	ENSMUSG00000016458	ENSMUST00000143043.8	8.431285
			ENSMUST00000111099.2	6.939276
			ENSMUST00000111098.8	5.066908
Zhx2	zinc fingers and homeoboxes 2	ENSMUSG00000071757	ENSMUST00000096430.11	7.701803
Gapdh	glyceraldehyde-3-phosphate dehydrogenase	ENSMUSG00000057666	ENSMUST00000118875.8	4856,813242
			ENSMUST00000117757.9	2833,490407
			ENSMUST00000073605.15	813,747632

[1] For the comparison of transcript levels of the listed genes, the expression of the housekeeping gene glyceraldehyde-3-phosphate dehydrogenase (*Gapdh*) is shown. The complete mRNA expression profile of Col-GFP HSC cells, as observed by NGS, can be found in Table S5. ND indicates that no transcripts of this gene were detected. TPM, Transcripts Per Million.

Table S7. Selected Kupffer cell marker gene expression in Col-GFP HSCs

Gene	Gene description	Gene Id	Transcript Id	TPM[1]
Adgre1	adhesion G protein-coupled receptor E1 (F4/80)	ENSMUSG00000004730	ND	0
Adgre4	adhesion G protein-coupled receptor E4	ENSMUSG00000032915	ND	0
C1qa	complement component 1, q subcomponent, alpha polypeptide	ENSMUSG00000036887	ND	0
C1qb	complement component 1, q subcomponent, beta polypeptide	ENSMUSG00000036905	ND	0
Ccr5	chemokine (C-C motif) receptor 5	ENSMUSG00000079227	ND	0
Chit1	chitinase 1 (chitotriosidase)	ENSMUSG00000026450	ND	0
Cd14	CD14 antigen	ENSMUSG00000051439	ENSMUST00000061829.8	2.436735
Cd38	CD38 antigen	ENSMUSG00000029084	ND	0
Cd163	CD163 antigen	ENSMUSG00000008845	ND	0
Clec1b	C-type lectin domain family 1, member b	ENSMUSG00000030159	ND	0
Clec4e	C-type lectin domain family 4, member e	ENSMUSG00000030142	ND	0
Clec4f	C-type lectin domain family 4, member f	ENSMUSG00000014542	ND	0
Clec4g	C-type lectin domain family 4, member g	ENSMUSG00000074491	ENSMUST00000062037.7	0.420295
Csf1r	colony stimulating factor 1 receptor	ENSMUSG00000024621	ENSMUST00000025523.13	0.360052
Dnase1l3	deoxyribonuclease 1-like 3	ENSMUSG00000025279	ND	0
Ear2	eosinophil-associated, ribonuclease A family, member 2	ENSMUSG00000072596	ND	0
Fcna	ficolin A	ENSMUSG00000026938	ND	0
Folr2	folate receptor 2 (fetal)	ENSMUSG00000032725	ND	0
Gpihbp1	GPI-anchored HDL-binding protein 1	ENSMUSG00000022579	ND	0
Il1b	interleukin 1 beta	ENSMUSG00000027398	ND	0
Irf7	interferon regulatory factor 7	ENSMUSG00000025498	ENSMUST00000106023.8	1.942536
Marco	macrophage receptor with collagenous structure	ENSMUSG00000026390	ND	0
Mndal	myeloid nuclear differentiation antigen like	ENSMUSG00000090272	ENSMUST00000186442.7<br>ENSMUST00000188804.7	0.951943<br>0.464614
Mpo	myeloperoxidase	ENSMUSG00000009350	ND	0
Msr1	macrophage scavenger receptor 1	ENSMUSG00000025044	ND	0
Osm	oncostatin M	ENSMUSG00000058755	ND	0
Pltp	phospholipid transfer protein	ENSMUSG00000017754	ENSMUST00000059954.14<br>ENSMUST00000109316.8	41.660373<br>4.203783
Ppara	peroxisome proliferator activated receptor alpha	ENSMUSG00000022383	ND	0
Ppard	peroxisome proliferator activator receptor delta	ENSMUSG00000002250	ENSMUST00000002320.16	29.475367
Prok2	prokineticin 2	ENSMUSG00000030069	ND	0
Siglecf	sialic acid binding Ig-like lectin F	ENSMUSG00000039013	ND	0
Slc11a1	solute carrier family 11 (proton-coupled divalent metal ion transporters), member 1	ENSMUSG00000026177	ND	0
Slc15a3	solute carrier family 15, member 3	ENSMUSG00000024737	ND	0

Slc40a1	solute carrier family 40 (iron-regulated transporter), member 1	ENSMUSG00000025993	ENSMUST00000027137.11	3.194646
Slc41a1	solute carrier family 41, member 1	ENSMUSG00000013275	ENSMUST00000086559.7	9.994692
Stard5	StAR-related lipid transfer (START) domain containing 5	ENSMUSG00000046027	ENSMUST00000075418.15	5.972625
Timd4	T cell immunoglobulin and mucin domain containing 4	ENSMUSG00000055546	ND	0
Tlr4	toll-like receptor 4	ENSMUSG00000039005	ENSMUST00000048096.12	10.680585
Tlr9	toll-like receptor 9	ENSMUSG00000045322	ND	0
Tnf	tumor necrosis factor	ENSMUSG00000024401	ND	0
Trem1	triggering receptor expressed on myeloid cells 1	ENSMUSG00000042265	ND	0
Vdr	vitamin D (1,25-dihydroxyvitamin D3) receptor	ENSMUSG00000022479	ENSMUST00000023119.15	1.901802
Vsig4	V-set and immunoglobulin domain containing 4	ENSMUSG00000044206	ND	0
Gapdh	glyceraldehyde-3-phosphate dehydrogenase	ENSMUSG00000057666	ENSMUST00000118875.8<br>ENSMUST00000117757.9<br>ENSMUST00000073605.15	4856,813242<br>2833,490407<br>813,747632

[1] For the comparison of transcript levels of the listed genes, the expression of the housekeeping gene glyceraldehyde-3-phosphate dehydrogenase (*Gapdh*) is shown. The complete mRNA expression profile of Col-GFP HSC cells, as observed by NGS, can be found in Table S5. ND indicates that no transcripts of this gene were detected. TPM, Transcripts Per Million.

International Journal of
Molecular Sciences

Table S8. Selected liver sinusoidal endothelial cell marker gene expression in Col-GFP HSCs

Gene	Gene description	Gene Id	Transcript Id	TPM[1]
Cd32B	Fc receptor, IgG, low affinity IIb	ENSMUSG00000026656	ND	0
Cd36	CD36 molecule	ENSMUSG00000002944	ND	0
Clec1B	C-type lectin domain family 1, member b	ENSMUSG00000030159	ND	0
Eng	Endoglin (CD105)	ENSMUSG00000026814	ENSMUST00000009705.14	24,094436
			ENSMUST00000113272.8	20,652491
			ENSMUST00000167841.8	2,10756
Icam1	intercellular adhesion molecule 1 (CD54)	ENSMUSG00000037405	ND	0
Lyve1	lymphatic vessel endothelial hyaluronan receptor 1	ENSMUSG00000030787	ND	0
Mcam	melanoma cell adhesion molecule (CD146)	ENSMUSG00000032135	ENSMUST00000034650.15	34,189753
			ENSMUST00000098852.3	13,06351
Pecam-1	platelet/endothelial cell adhesion molecule 1 (CD31)	ENSMUSG00000020717	ND	0
Ptprc	protein tyrosine phosphatase receptor type C (CD45)	*ENSMUSG00000026395*	ND	0
Stab1	stabilin 1	ENSMUSG00000042286	ND	0
Stab2	stabilin 2	ENSMUSG00000035459	ND	0
Vvf	Von Willebrand factor	ENSMUSG00000001930	ND	0
Gapdh	glyceraldehyde-3-phosphate dehydrogenase	ENSMUSG00000057666	ENSMUST00000118875.8	4856,813242
			ENSMUST00000117757.9	2833,490407
			ENSMUST00000073605.15	813,747632

[1] For the comparison of transcript levels of the listed genes, the expression of the housekeeping gene glyceraldehyde-3-phosphate dehydrogenase (*Gapdh*) is shown. The complete mRNA expression profile of Col-GFP HSC cells, as observed by NGS, can be found in Table S5. ND indicates that no transcripts of this gene were detected. TPM, Transcripts Per Million.

International Journal of *Molecular Sciences*

Table S9. Selected cholangiocyte marker gene expression in Col-GFP HSCs

Gene	Gene description	Gene Id	Transcript Id	TPM[1]
Alb	albumin	ENSMUSG00000029368	ENSMUST00000031314.10	2.156572
Agr2	anterior gradient 2	ENSMUSG00000020581	ND	0
Alpl	alkaline phosphatase, liver/bone/kidney	ENSMUSG00000028766	ND	0
Aqp1	aquaporin 1	ENSMUSG00000004655	ENSMUST00000004774.4	1.812733
Aqp4	aquaporin 4	ENSMUSG00000024411	ND	0
Cckbr	cholecystokinin B receptor	ENSMUSG00000030898	ND	0
Cftr	cystic fibrosis transmembrane conductance regulator	ENSMUSG00000041301	ENSMUST00000045706.12	0.288949
Cldn4	claudin 4	ENSMUSG00000047501	ND	0
Cxcl1	chemokine (C-X-C motif) ligand 1	ENSMUSG00000029380	ENSMUST00000031327.9	0.579824
Defb1	defensin beta 1	ENSMUSG00000044748	ND	0
Elf3	E74-like factor 3	ENSMUSG00000003051	ND	0
Epcam	epithelial cell adhesion molecule	ENSMUSG00000045394	ND	0
Fxyd2	FXYD domain-containing ion transport regulator 2	ENSMUSG00000059412	ND	0
Ggt1	gamma-glutamyltransferase 1	ENSMUSG00000006345	ENSMUST00000134503.8	0.226583
Ggt6	gamma-glutamyltransferase 6	ENSMUSG00000040471	ND	0
Ggt7	gamma-glutamyltransferase 7	ENSMUSG00000027603	ENSMUST00000029131.11	3.827169
Gpbar1	G protein-coupled bile acid receptor 1	ENSMUSG00000064272	ND	0
Hnf1b	HNF1 homeobox B	ENSMUSG00000020679	ND	0
Itgb4	integrin beta 4	ENSMUSG00000020758	ENSMUST00000106460.9	0.231242
Jag1	jagged 1	ENSMUSG00000027276	ENSMUST00000028735.8	2.199048
Kcnn2	potassium intermediate/small conductance calcium-activated channel, subfamily N, member 2	ENSMUSG00000054477	ENSMUST00000183850.8	1.710928
Krt7	keratin 7	ENSMUSG00000023039	ENSMUST00000068904.9	0.113128
Krt19	keratin 19	ENSMUSG00000020911	ENSMUST00000007317.8	89.927899
Lgals2	lectin, galactose-binding, soluble 2	ENSMUSG00000043501	ENSMUST00000044584.6	1.101417
Lgals4	lectin, galactose binding, soluble 4	ENSMUSG00000053964	ND	0
Lcn2	lipocalin 2	ENSMUSG00000026822	ND	0
Mmp7	matrix metallopeptidase 7	ENSMUSG00000018623	ND	0
Muc5b	mucin 5, subtype B, tracheobronchial	ENSMUSG00000066108	ND	0
Onecut2	one cut domain, family member 2	ENSMUSG00000045991	ENSMUST00000175965.10	0.082557
Pigr	polymeric immunoglobulin receptor	ENSMUSG00000026417	ND	0
Scgb3a1	secretoglobin, family 3A, member 1	ENSMUSG00000064057	ND	0
Sctr	secretin receptor	ENSMUSG00000026387	ND	0
Sox9	SRY (sex determining region Y)-box 9	ENSMUSG00000000567	ENSMUST00000000579.3	0.783664
Spp1	secreted phosphoprotein 1	ENSMUSG00000029304	ENSMUST00000031243.15	9.420875
Sstr2	somatostatin receptor 2	ENSMUSG00000047904	ENSMUST00000067591.3	0.519755
Tacstd2	tumor-associated calcium signal transducer 2	ENSMUSG00000051397	ND	0
Tff1	trefoil factor 1	ENSMUSG00000024032	ND	0
Tff2	trefoil factor 2 (spasmolytic protein 1)	ENSMUSG00000024028	ND	0

International Journal of
Molecular Sciences

Tff3	trefoil factor 3, intestinal	ENSMUSG00000024029	ND	0
Gapdh	glyceraldehyde-3-phosphate dehydrogenase	ENSMUSG00000057666	ENSMUST00000118875.8	4856,813242
			ENSMUST00000117757.9	2833,490407
			ENSMUST00000073605.15	813,747632

[1] For the comparison of transcript levels of the listed genes, the expression of the housekeeping gene glyceraldehyde-3-phosphate dehydrogenase (*Gapdh*) is shown. The complete mRNA expression profile of Col-GFP HSC cells, as observed by NGS, can be found in Table S5. ND indicates that no transcripts of this gene were detected. TPM, Transcripts Per Million.

International Journal of Molecular Sciences

Table S10. Primers used in this study (in alphabetical order)

Gene	Accession No.	Gene lenghts (bp)	Forward primer (5'->3')	Location	Reverse primer (5'->3')	Location	Amplicon size (bp)	Method	Source
Acta2	NM_007392.3	2572	tgc tgg act ctg gag atg	568-585	gtg atc acc tgc ccg tc	859-843	292	RT-PCR	[6]
Actb	NM_007393.5	1935	ctc tag act tcg agc agg aga tgg	768-791	atg cca cag gat tcc ata ccc aag a	930-906	163	RT-PCR, RT-qPCR	[7]
Actn1	NM_134156.3	3731	gat att ggc aac gac ccc ca	2613-2632	agc ggt tgg ggt cta caa tg	2682-2663	70	RT-qPCR	[8]
Arpc2	NM_029711.3	1599	tcc ttc ccc tgc ccg	254-268	act tct aca gct tct ggt ttg t	380-359	127	RT-qPCR	[8]
Alb	NM_009654.4	2076	ggt ctc atc tgt ccg tca gag	707-727	gga agg aca tcc ttg gcc tca g	1117-1096	411	RT-PCR	[8]
Ccnd1	NM_001379248.1	3768	agt gcg tgc aga agg aga tt	275-294	cac aac ttc tcg gca gtc aa	512-493	238	RT-qPCR	[9]
Ccl2	NM_011333.3	806	gtg ttg gct cag cca gat gc	146-165	gac acc tgc tgc tgg tga tcc	263-243	118	RT-qPCR	[10]
Cxcl5	NM_009141.3	1656	tgt cca caa tga gcc tcc ag	138-157	gct atg act gag gaa ggg gc	285-266	148	RT-qPCR	[8]
Foxa2	NM_001291065.1	2070	cat ccg act gga gca gct a	88-106	gcg ccc aca tag gat gac	265-248	178	RT-PCR	[11]
Fth1	NM_010239.2	942	tgg agt tgt atg cct cct acg	304-324	tgg aga aag tat ttg gca aag tt	397-375	94	RT-qPCR	[12]
Ftl1	NM_010240.2	986	cca gga tgt gca gaa gcc a	492-510	gcc ctt gga agg tac aga g	831-813	340	RT-qPCR	[8]
Gapdh	NM_001289726.2	1297	act gcc acc cag aag act g	650-668	cac cac cct gtt gct gta g	1081-1063	432	RT-PCR	[10]
Gapdh	NM_008084.4	1257	tgt gtc cgt cgt gga tct ga	783-802	ttg ctg ttg aag tcg cag gag	932-912	150	RT-qPCR	[13]
Got2	NM_010325.3	2385	atg gct gct gcc ttt cac	133-150	gat ctg gag gtc cca ttt ca	223-204	91	RT-qPCR	[14]
Hmgb1	NM_010439.4	2855	cac agc cat tgc agt aca ttg a	67-88	tgc ttg tca tct gct gca gtg t	597-576	531	RT-qPCR	[15]
Hnf4a	NM.008261.3	4393	agg caa tga cta cat cgt ccc	885-905	cag acc ctc cga gaa gca tc	1285-1266	401	RT-PCR	[16]
Hnf4a	NM_008261.3	4391	cca aga ggt cca tgg tgt tt	848-867	ccg agg gac gat gta gtc at	909-890	62	RT-qPCR	[8]
Lamp2	NM_010685.4	3651	aag gtg caa cct ttt aat gtg ac	1223-1245	tgt cat cat cca gcg aac ac	1292-1273	70	RT-qPCR	[17]
Myl6	NM_001317217.2	730	ctg cca gtg gct gac cat aa	534-553	cac tgg gca agg aac aga ct	621-602	88	RT-qPCR	[8]
Pcna	NM_011045.2	1260	tgc tct gag gta cct gaa ct	765-784	tgc ttc ctc atc ttc aat ct	924-905	160	RT-qPCR	[18]
Plin2	NM_007408.4	1680	ctc cac tcc act gtc cac ct	827-846	gct tat cct gag cac cct ga	911-892	85	RT-qPCR	[19]

International Journal of Molecular Sciences

Ppia	NM_008907.2	746	tgt gcc agg gtg gtg act tt	226-245	cgt ttg tgt ttg gtc cag cat	369-349	144	RT-qPCR	[20]
Prdx1	NM_011034.5	1416	tgc caa gtg att ggc gct tct g	284-305	agc aat ggt gcg ctt ggg atc t	412-391	129	RT-qPCR	[21]
Rpl13a	NM_009438.5	1039	tac cag aaa gtt tgc tta cct ggg	424-447	tgc ctg ttt ccg taa cct caa g	574-553	151	RT-qPCR	[22]
Rn18s	NR_003278.3	1870	ctc aac acg gga aac ctc ac	1247-1266	cgc tcc acc aac taa gcc cg	1356-1341	110	RT-PCR, RT-qPCR	[23]
Rps6	NM_009096.3	1394	ccc atg aag caa ggt gtt ct	225-244	aca atg cat cca cga aca ga	346-327	122	RT-qPCR	[10]
Slc40a1	NM_016917.2	3380	acc cat ccc cat agt ctc tgt	1672-1692	acc gtc aaa tca aag gac ca	1755-1736	84	RT-qPCR	[24]
Tfrc	NM_011638.4	4920	tgg gtc taa gtc tac agt ggc	2077-2097	aga tac ata ggg cga cag gaa	2246-2226	170	RT-qPCR	[25]
Tpi1	NM_009415.3	1469	aga ggg act cgg ggt gat	393-410	cct tgc tcc agt ctt tca ca	517-498	118	RT-qPCR	[8]
Tpm1	NM_001164248.1	1210	tcc agc tga aag agg cca ag	518-537	ctt ctg aga gct cag ccc g	643-625	126	RT-qPCR	[8]
Vdac1	NM_011694.7	1804	acc ttt gat tcg tca ttc tcg	370-390	tgc tcc ctc ttg tac cct gt	443-424	74	RT-qPCR	[26]
Vim	NM_011701.4	1834	cca acc ttt tct tcc ctg aac	1367-1387	ttg agt ggg tgt caa cca ga	1436-1417	70	RT-qPCR	[8]

International Journal of
Molecular Sciences

Table S11. Primary and secondary antibodies used for Western blot analysis (in alphabetical order)[1]

Antibody	Cat. no.	RRID[2]	Company	Size (kDa)	Dilution	Clonality
α-Actinin 1 (D6F6)	6487	AB_11179206	Cell Signaling Technology, Leiden, The Netherlands	100	1:1,000	r mAb
Albumin	4929S	AB_2225785	Cell Signaling Technology	67	1:1,000	r pAb
α-SMA (EPR5368)	ab124964	AB_11129103	Abcam, Cambridge, UK	43	1:1,000	m rAb
β-Actin (AC-15)	A5441	AB_476744	Sigma-Aldrich	42	1:10,000	m mAb
Caveolin-1	3238	AB_2072166	Cell Signaling Technology	21, 24	1:1,000	r pAb
Cyclin D1 (92G2)	2978S	AB_2259616	Cell Signaling Technology	36	1:1,000	r mAb
Collagen I	14695-1-AP	AB_2082037	Proteintech, Planegg-Martinsried, Germany	125	1:1,000	p rAb
Collagen IV	ab6586	AB_305584	Abcam	125	1:1,000	p rAb
Ferritin heavy chain 1 (D1D4)	4393	AB_11217441	Cell Signaling Technology	21	1:1,000	r mAb
Ferritin light chain 1	ab69090	AB_1523609	Abcam	21	1:1,000	r pAb
Fibronectin	AB1954	AB_2105708	Sigma-Aldrich	262	1:3,000	r pAb
GAPDH (6C5)	sc-32233	AB_627679	Santa Cruz Biotechnology, Santa Cruz, CA, USA	39	1:1,000	m mAb
GFP (FL)*	sc-8334	AB_641123	Santa Cruz Biotechnology	31	1:500	r pAb
HNF-4α (C-19)*	sc-6556	AB_2117025	Santa Cruz Biotechnology	54	1:1,000	g pAb
HSP90 (C45G5)	4877	AB_2233307	Cell Signaling Technology	90	1:1,000	r mAb
PCNA (6D645)	sc-71858	AB_1127080	Santa Cruz Biotechnology	36	1:1,000	m mAb
SV40T Ag (v-300)*	sc-20800	AB_661493	Santa Cruz Biotechnology	92	1:1,000	r pAb
Vimentin (EPR3776)	ab92547	AB_10562134	Abcam	53	1:3,000	r mAb
Goat anti-rabbit IgG (H+L), HRP	31460	AB_228341	ThermoFisher Scientific, Schwerte, Germany	NA	1:5,000	g pAb
Goat anti-mouse IgG (H+L), HRP	31430	AB_228307	ThermoFisher Scientific	NA	1;5,000	g pAb
Mouse anti-goat IgG (H+L), HRP	31400	AB_228370	ThermoFisher Scientific	NA	1:5,000	m pAb

[1]Abbreviations used are: g, goat; m, mouse; mAb, antibody; pAb, polyclonal antibody monoclonal; r, rabbit; [2]Data were taken from the Research Resource Identifier (RRID) portal, which is available at https://www.rrids.org/. * Distribution of this antibody has been discontinued.

References to Supplements

1. Meurer SK, Alsamman M, Sahin H, Wasmuth HE, Kisseleva T, Brenner DA, Trautwein C, Weiskirchen R, Scholten D. Overexpression of endoglin modulates TGF-β1-signalling pathways in a novel immortalized mouse hepatic stellate cell line. PLoS One 2013;8(2):e56116. doi: 10.1371/journal.pone.0056116
2. Stewart TJ, Abrams SI. Altered immune function during long-term host-tumor interactions can be modulated to retard autochthonous neoplastic growth. J Immunol. 2007;179(5):2851-9. doi: 10.4049/jimmunol.179.5.2851
3. Murphy BM, Jensen DM, Arnold TE, Aguilar-Valenzuela R, Hughes J, Posada V, Nguyen KT, Chu VT, Tsai KY, Burd CJ, Burd CE. The OSUMMER lines: A series of ultraviolet-accelerated NRAS-mutant mouse melanoma cell lines syngeneic to C57BL/6. Pigment Cell Melanoma Res. 2023;36(5):365-377. doi: 10.1111/pcmr.13107
4. Meeth K, Wang JX, Micevic G, Damsky W, Bosenberg MW. The YUMM lines: a series of congenic mouse melanoma cell lines with defined genetic alterations. Pigment Cell Melanoma Res. 2016;29(5):590-7. doi: 10.1111/pcmr.12498
5. Wang J, Perry CJ, Meeth K, Thakral D, Damsky W, Micevic G, Kaech S, Blenman K, Bosenberg M. UV-induced somatic mutations elicit a functional T cell response in the YUMMER1.7 mouse melanoma model. Pigment Cell Melanoma Res. 2017;30(4):428-435. doi: 10.1111/pcmr.12591
6. Kordes C, Sawitza I, Müller-Marbach A, Ale-Agha N, Keitel V, Klonowski-Stumpe H, Häussinger D. CD133+ hepatic stellate cells are progenitor cells. Biochem Biophys Res Commun. 2007;352(2):410-7. doi: 10.1016/j.bbrc.2006.11.029
7. Borkham-Kamphorst E, van de Leur E, Zimmermann HW, Karlmark KR, Tihaa L, Haas U, Tacke F, Berger T, Mak TW, Weiskirchen R. Protective effects of lipocalin-2 (LCN2) in acute liver injury suggest a novel function in liver homeostasis. Biochim Biophys Acta 2013;1832(5):660-73. doi: 10.1016/j.bbadis.2013.01.014
8. PCR Primer Design. Available at: https://eurofinsgenomics.eu/en/ecom/tools/pcr-primer-design/ (last accessed 23 June 2025)
9. Oh HR, Kim J, Kim J. Critical roles of Cyclin D1 in mouse embryonic fibroblast cell reprogramming. FEBS J. 2016;283(24):4549-68. doi: 10.1111/febs.13941
10. Boaru SG, Borkham-Kamphorst E, Tihaa L, Haas U, Weiskirchen R. Expression analysis of inflammasomes in experimental models of inflammatory and fibrotic liver disease. J Inflamm (Lond). 2012;9(1):49. doi: 10.1186/1476-9255-9-49
11. Kofent J, Zhang J, Spagnoli FM. The histone methyltransferase Setd7 promotes pancreatic progenitor identity. Development 2016;143(19):3573-81. doi: 10.1242/dev.136226
12. Ito J, Omiya S, Rusu MC, Ueda H, Murakawa T, Tanada Y, Abe H, Nakahara K, Asahi M, Taneike M, Nishida K, Shah AM, Otsu K. Iron derived from autophagy-mediated ferritin degradation induces cardiomyocyte death and heart failure in mice. Elife 2021;10:e62174. doi: 10.7554/eLife.62174
13. Zhang J, Liu Y, Liu X, Li S, Cheng C, Chen S, Le W. Dynamic changes of CX3CL1/CX3CR1 axis during microglial activation and motor neuron loss in the spinal cord of ALS mouse model. Transl Neurodegener. 2018;7:35. doi: 10.1186/s40035-018-0138-4
14. Fan L, Lesser AF, Sweet DR, Keerthy KS, Lu Y, Chan ER, Vinayachandran V, Ilkayeva O, Das T, Newgard CB, Jain MK. KLF15 controls brown adipose tissue transcriptional flexibility and metabolism in response to various energetic demands. iScience 2022;25(11):105292. doi: 10.1016/j.isci.2022.105292
15. Huang H, Nace GW, McDonald KA, Tai S, Klune JR, Rosborough BR, Ding Q, Loughran P, Zhu X, Beer-Stolz D, Chang EB, Billiar T, Tsung A. Hepatocyte-specific high-mobility group box 1 deletion worsens the injury in liver ischemia/reperfusion: a role for intracellular high-mobility group box 1 in cellular protection. Hepatology 2014;59(5):1984-97. doi: 10.1002/hep.26976

16. Park MR, Wong MS, Araúzo-Bravo MJ, Lee H, Nam D, Park SY, Seo HD, Lee SM, Zeilhofer HF, Zaehres H, Schöler HR, Kim JB. Oct4 and Hnf4α-induced hepatic stem cells ameliorate chronic liver injury in liver fibrosis model. PLoS One 2019;14(8):e0221085. doi: 10.1371/journal.pone.0221085
17. Tanaka Y, Watari M, Saito T, Morishita Y, Ishibashi K. Enhanced autophagy in polycystic kidneys of AQP11 null mice. Int J Mol Sci. 2016;17(12):1993. doi: 10.3390/ijms17121993
18. Lee JY, Khan AA, Min H, Wang X, Kim MH. Identification and characterization of a noncoding RNA at the mouse Pcna locus. Mol Cells 2012;33(2):111-6. doi: 10.1007/s10059-012-2164-x
19. Asimakopoulou A, Vucur M, Luedde T, Schneiders S, Kalampoka S, Weiss TS, Weiskirchen R. Perilipin 5 and lipocalin 2 expression in hepatocellular carcinoma. Cancers (Basel) 2019;11(3):385. doi: 10.3390/cancers11030385
20. Gong ZK, Wang SJ, Huang YQ, Zhao RQ, Zhu QF, Lin WZ. Identification and validation of suitable reference genes for RT-qPCR analysis in mouse testis development. Mol Genet Genomics 2014;289(6):1157-69. doi: 10.1007/s00438-014-0877-6
21. Dong Z, Bian L, Wang YL, Sun LM. Gastrodin protects against high glucose-induced cardiomyocyte toxicity via GSK-3β-mediated nuclear translocation of Nrf2. Hum Exp Toxicol. 2021;40(9):1584-97. doi: 10.1177/09603271211002885
22. Schroder AL, Pelch KE, Nagel SC. Estrogen modulates expression of putative housekeeping genes in the mouse uterus. Endocrine. 2009;35(2):211-9. doi: 10.1007/s12020-009-9154-6
23. Lin P, Lan X, Chen F, Yang Y, Jin Y, Wang A. Reference gene selection for real-time quantitative PCR analysis of the mouse uterus in the peri-implantation period. PLoS One 2013;8(4):e62462. doi: 10.1371/journal.pone.0062462
24. Schröder SK, Krizanac M, Kim P, Kessel JC, Weiskirchen R. Ovaries of estrogen receptor 1-deficient mice show iron overload and signs of aging. Front Endocrinol (Lausanne) 2024;15:1325386. doi: 10.3389/fendo.2024.1325386
25. Ma J, Ma HM, Shen MQ, Wang YY, Bao YX, Liu Y, Ke Y, Qian ZM. The role of Iron in atherosclerosis in apolipoprotein E deficient mice. Front Cardiovasc Med. 2022;9:857933. doi: 10.3389/fcvm.2022.857933
26. Faragó N, Kocsis GF, Fehér LZ, Csont T, Hackler L Jr, Varga-Orvos Z, Csonka C, Kelemen JZ, Ferdinandy P, Puskás LG. Gene and protein expression changes in response to normoxic perfusion in mouse hearts. J Pharmacol Toxicol Methods 2008;57(2):145-54. doi: 10.1016/j.vascn.2008.01.001
